# Enhanced Performance of a Monolayer MoS_2_/WSe_2_ Heterojunction as a Photoelectrochemical Cathode

**DOI:** 10.1007/s40820-018-0212-6

**Published:** 2018-07-03

**Authors:** Jingwei Xiao, Yu Zhang, Huanjun Chen, Ningsheng Xu, Shaozhi Deng

**Affiliations:** State Key Laboratory of Optoelectronic Materials and Technologies, Guangdong Province Key Laboratory of Display Material and Technology, School of Electronics and Information Technology, Sun Yat-sen University, Guangzhou, 510275 People’s Republic of China

**Keywords:** MoS_2_/WSe_2_, Monolayer, Bilayer, Heterojunction, Photoelectrochemical cathode

## Abstract

Transition-metal dichalcogenide (TMD) semiconductors have attracted interest as photoelectrochemical (PEC) electrodes due to their novel band-gap structures, optoelectronic properties, and photocatalytic activities. However, the photo-harvesting efficiency still requires improvement. In this study, A TMD stacked heterojunction structure was adopted to further enhance the performance of the PEC cathode. A *P*-type WSe_2_ and an *N*-type MoS_2_ monolayer were stacked layer-by-layer to build a ultrathin vertical heterojunction using a micro-fabrication method. In situ measurement was employed to characterize the intrinsic PEC performance on a single-sheet heterostructure. Benefitting from its built-in electric field and type II band alignment, the MoS_2_/WSe_2_ bilayer heterojunction exhibited exceptional photocatalytic activity and a high incident photo-to-current conversion efficiency (IPCE). Comparing with the monolayer WSe_2_ cathode, the PEC current and the IPCE of the bilayer heterojunction increased by a factor of 5.6 and enhanced 50%, respectively. The intriguing performance renders the MoS_2_/WSe_2_ heterojunction attractive for application in high-performance PEC water splitting.
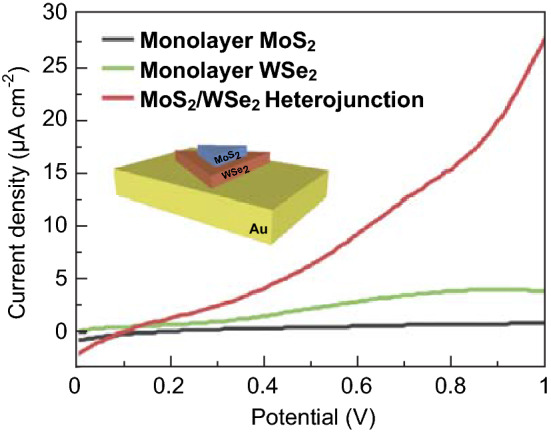

## Highlights

- A vertical transition-metal dichalcogenide MoS_2_/WSe_2_ bilayer heterojunction was built by stacking a *P*-type WSe_2_ and an *N*-type MoS_2_ monolayer.
- An in situ measurement method was employed to characterize the intrinsic photoelectrochemical performance on the microscale.
- The photoelectrochemical current and the incident photo-to-current conversion efficiency of the MoS_2_/WSe_2_ bilayer heterojunction increased by a factor of 5.6 and enhanced 50% compared with the monolayer WSe_2_ cathode.

## Introduction

Hydrogen-based energy is a clean, sustainable, and highly efficient energy resource. Intensive research has been conducted to realize efficient production of hydrogen, and photoelectrochemical (PEC) water splitting is considered as a promising method [1–5]. A high-performance electrode material that can fully utilize solar energy and efficiently run water redox reactions is the key to PEC application. Recently, transition-metal dichalcogenide (TMD) semiconductors (MoS_2_, WSe_2_, WS_2_, etc.) have been proposed as candidates for PEC electrode materials due to their novel band-gap structures, superior electrochemical properties and low cost [5, 6]. When the thickness of TMDs is varied from bulk to single layer, which become two-dimensional (2D) materials, their band gaps change from indirect (1.0–1.6 eV) to direct (1.6–1.8 eV) [7]. 2D material is a kind of layered material that consists of single or few atomic layers, such as graphene. The 2D material family contains carbon material, TMDs and layered metal oxides, etc. [8]. The change of band gaps allows 2D TMDs to absorb visible light, thus improving the photoconversion efficiency [9–11]. Additionally, layered TMDs have high absorption coefficients, permitting the absorption of 5–10% of incident sunlight by monolayer TMD [12]. All these features make layered TMDs attractive materials for solar-driven water-splitting devices.

In the group of TMDs, WSe_2_ and MoS_2_ are the choices with the best properties. *P*-type WSe_2_ has been identified as an active and promising electrocatalyst for the hydrogen evolution reaction (HER) [13, 14]. Monolayer WSe_2_ has a direct band gap of ~ 1.65 eV, which corresponds to ~ 750 nm wavelength light, and exhibits a high hole mobility (~ 140 cm^2^ V^−1^ s^−1^) that is suitable for PEC cathodes [13, 15, 16]. Recent reports have demonstrated favorable PEC properties of WSe_2_. A large-area WSe_2_ flake Pt-decorating thin film fabricated using a space-confined self-assembled thin film deposition method demonstrated good PEC performance [14].

*N*-type MoS_2_ has also been demonstrated to be an active catalyst in photocatalytic reactions [4, 17, 18]. Monolayer MoS_2_ has a direct band gap of 1.85 eV and an electron mobility of 200 cm^2^ V^−1^ s^−1^ [19]. Yu et al. reported its catalytic activity for hydrogen evolution [20], and Chen et al. and King et al. reported its application using silicon as a photocathode for PEC water splitting [21, 22].

Nevertheless, the PEC efficiency of few-layer or single-layer WSe_2_ as a photocathode is still limited. To further improve the efficiency, coupling WSe_2_ with a MoS_2_ monolayer sheet to form a heterojunction could be an ideal choice. Owing to the suitable band gap and band position of the components, it has been reported that the MoS_2_/WSe_2_ heterojunction can be applied as a high-performance *p*–*n* diode [23] and transistor [24, 25]. As a PEC cathode, the advantages of the TMD heterojunction are that: (1) the built-in field in the depletion layer of the *p*–*n* junction may accelerate separation of the photo-generated excitons, as well as restrict recombination of the electron–hole pair to improve the PEC performance [26–28]; (2) the atom-thin vertical heterojunction could shorten the diffusion distance and rapidly deliver the excitons to the solid–liquid interface for redox reaction [17, 29]; (3) due to the large contact area in the heterojunction, more charge could be efficiently separated simultaneously; (4) an extended region of the visible-light spectrum could be utilized by this MoS_2_/WSe_2_ heterojunction.

In this study, we fabricated a 2D MoS_2_/WSe_2_ heterojunction PEC cathode and demonstrated its improved PEC performance. A micro-fabrication method is adopted to build a single-sheet stacked bilayer heterostructure. In situ measurement is employed to characterize the intrinsic PEC performance of the micro-heterostructure. The mechanism of enhancement of the PEC characteristics of the 2D heterojunction is also discussed.

## Experimental

We fabricated a single-sheet MoS_2_/WSe_2_ heterojunction PEC device on the microscale and adopted an in situ measurement technique to characterize its performance. This is an advance method of characterizing the intrinsic PEC characteristics of heterojunction devices due to the unique material properties of a single sheet. Most of the interference factors such as the grain boundary, defects and inhomogeneity are eliminated in the single-sheet device by using in situ measurement.

### Synthesis and Transfer of PEC Cathode Materials

Monolayer WSe_2_ and MoS_2_ were firstly synthesized on respective sapphire and SiO_2_/Si substrate using chemical vapor deposition. The WSe_2_ and MoS_2_ sheets were then transferred to the PEC Au cathode on a silicon substrate to form the heterojunction. Specifically, polystyrene (PS) was first spin-coated onto the sapphire substrate and the substrate was then immersed in deionized water. The PS film with the WSe_2_ sheet was peeled off from the substrate due to its hydrophobicity and then pasted on a bulk polydimethylsiloxane (PDMS). Using a microscope platform, the WSe_2_ sheet on PDMS could be located and shifted to the top of the target Au electrode. WSe_2_ with the PS layer was heated for exfoliation from PDMS and transferred to the Au electrode. Finally, the PS was removed using methylbenzene, leaving the exfoliated WSe_2_ sheet on the target electrode. The material characteristics were confirmed from the Raman and photoluminescence (PL) spectra (RENISHAW, 532 nm laser, 70 μW incident power) and atomic force microscopy (AFM) (NT-MDT NTEGRA Spectra). The light absorption spectra of monolayer MoS_2_, WSe_2_ and MoS_2_/WSe_2_ heterojunction were measured by HITACHI U-4100 spectrophotometer.

### Fabrication of Devices

**Fig. 1:**
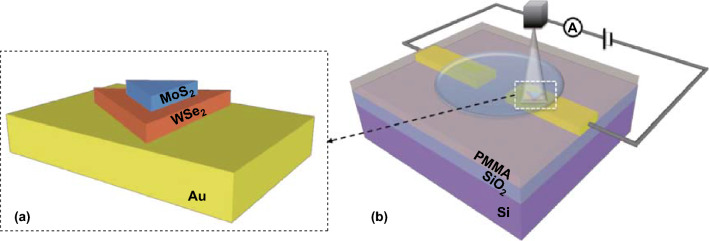
Schematic of **a** the MoS_2_/WSe_2_ heterojunction and **b** PEC device and measurement

The single-sheet PEC device was fabricated using a micro-fabrication and transfer method. A 50 m wide Au thin film was sputtered onto a Si substrate with a 300 nm oxide layer, as the cathode. A WSe_2_ flake was first transferred onto the Au electrode as the bottom layer of the heterojunction. The MoS_2_ flake was transferred on top of the WSe_2_ flake to form a vertical heterojunction (Fig. 1a). Another Au electrode on the other side of the substrate acted as the anode. The gap between the anode and cathode was 1.5 mm. Thereafter, the whole substrate was coated with photoresist, except for the area of the target heterojunction and the Au anode. This ensured that only the heterojunction and Au electrode were exposed to the solution and blocked the background noise. Two Al wires were, respectively, bonded onto the two Au electrode pads to output the PEC signal and connect the external measurement circuit (Fig. 1b).

### PEC Measurement

An optical microscope (Olympus BX53) with a high-power mercury lamp (U-RFL-T, 100 W) was used as a PEC measurement platform. The device was steadily fixed on the sample stage of the microscope. The PEC measurement circuit of the device is shown in Fig. 1b. A Keithley 2600 Dual-Channel System Source Meter was used to apply a bias voltage and measure the current. The MoS_2_/WSe_2_ heterojunction connected to the negative pole of the power source acted as the cathode, while the Au anode was connected to the positive pole. A droplet of electrolyte (0.5 mol L^−1^ Na_2_SO_4_ solution) was injected to cover the whole device. During the measurement, white light from a mercury lamp, simulating solar power, was used to illuminate the device. The illuminated area was controlled to as small as 0.2 mm in diameter by the pinhole of the microscope. An external voltage was applied to the working electrode and swept from 0 to 1 V (100 mV s^−1^), and the PEC current was recorded. To evaluate the relation of the PEC current to the visible-light spectrum, monochromatic light was separated from the white light using several optical filters (THORLABS, Optical bandwidth 10 nm). Optical filters were placed into the light path to select a specific wavelength. An optical power meter (GENTEC-EO UNO) was used to measure the incident light power of the different wavelengths, and a spot analyzer was used to confirm the size of the light spot.

## Results and Discussion

### Raman and PL Spectra of Monolayer Heterojunction

**Fig. 2:**
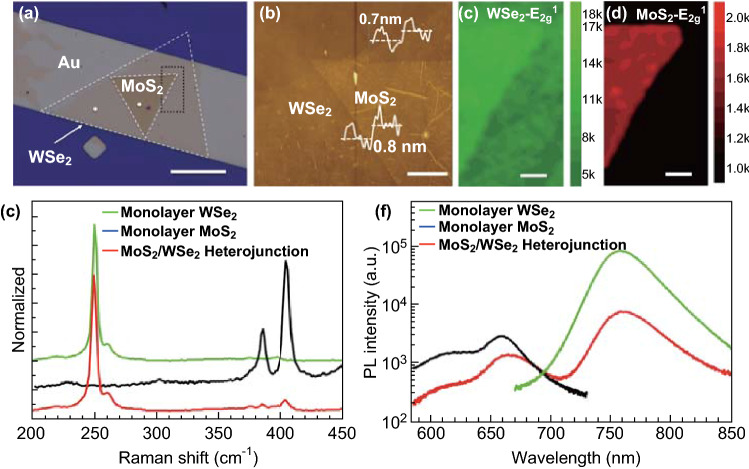
**a** Optical image of monolayer MoS_2_/WSe_2_ heterojunction (dashed in white triangle). The white dots marked in the optical image are the spots where the spectra are accumulated (scale bar is 30 m). **b** AFM scanning showing thickness of surface of MoS_2_/WSe_2_ heterojunction and line scan profile (scale bar is 4 m). **c**, **d** Raman intensity map of the WSe_2_ *E*_2g_^1^ mode (green) and MoS_2_ *E*_2g_^1^ mode (red) in heterostructure, corresponding to black dashed square area in (**a**) (scale bar is 5 m). **e**, **f** Raman and PL spectra of monolayer MoS_2_ (black line), WSe_2_ (green line) and MoS_2_/WSe_2_ heterojunction (red line). (Color figure online)

The optical image of the monolayer MoS_2_/WSe_2_ heterojunction is shown in Fig. 2a. A triangular MoS_2_ sheet was stacked on a larger triangular WSe_2_. These two stacked monolayer sheets formed a vertical heterojunction. The AFM showed that the step heights of monolayer WSe_2_ on the Au electrode and that of monolayer MoS_2_ on WSe_2_ were 0.7 and 0.8 nm, respectively, which confirmed the monolayer thickness of the WSe_2_ and MoS_2_ sheet (Fig. 2b).

The Raman and PL spectra of the mono-MoS_2_, WSe_2_, and MoS_2_/WSe_2_ heterojunction were acquired to characterize their crystallinity. Figure 2e shows the Raman spectra of the above three materials. Monolayer WSe_2_ has showed two strong peaks around 250 cm^−1^ corresponding to the *E*_2g_^1^ (in-plane) and *A*_1g_ (out-of-plane) modes. The Raman *B*_2g_^1^ mode at 310 cm^−1^ was not observed, which confirmed the monolayer sheet structure [30–32]. Monolayer MoS_2_ showed characteristic *E*_2g_^1^ and *A*_1g_ Raman mode signals at 385.0 and 405.6 cm^−1^, consistent with published reports [33–35] (Fig. 2e, black line). The Raman spectrum of the MoS_2_/WSe_2_ heterojunction showed all the peaks of single-layer WSe_2_ and MoS_2_. The peak intensity of WSe_2_ was much stronger than MoS_2_ [35]; therefore the Raman peak intensity of MoS_2_ obtained in heterojunction looks weaker. Actually, the Raman peak intensity of MoS_2_ in the heterojunction is the same as that of single-layer MoS_2_. The Raman mapping is used to further verify the crystal homogeneity of MoS_2_ and WSe_2_ in their heterostructure, as shown in Fig. 2c, d. The Raman intensity mapping used the *E*_2g_^1^ mode of both WSe_2_ and MoS_2_, which the color scale bar represents the intensity, respectively. In the overlapping area, the Raman intensity of WSe_2_ was stronger. The reason is related to the heterojunction stacking that active the Raman features.

The PL spectrum for monolayer WSe_2_ in Fig. 2f shows a strong single PL peak around 760 nm, nearly 1.63 eV, corresponding to the “A” exciton peak (Fig. 2e, green line). The strong emission and single symmetric PL peak at ~ 1.60 eV suggest the direct band-gap nature of monolayer WSe_2_ [33, 35, 36]. It is reported that the PL spectrum of multilayer WSe_2_ shows the “A” exciton peak and an additional broad peak at ~ 885 nm (call as “I” peak), which is attributed to indirect band-gap emission [36, 37]. Single-layer MoS_2_ showed a peak at 670 nm, nearly 1.85 eV, corresponding to “A” exciton. The PL yield of WSe_2_ was much higher than that of MoS_2_, suggesting stronger nonradiative recombination in the latter. The PL peak of WSe_2_ in heterostructure was about ten times lower than that of the individual WSe_2_. Such a significant quenching effect indicated that many photo-generated charge carriers were transferred from WSe_2_ to MoS_2_ [25, 33, 35, 36].

### PEC Performance of Bilayer Heterojunction

**Fig. 3:**
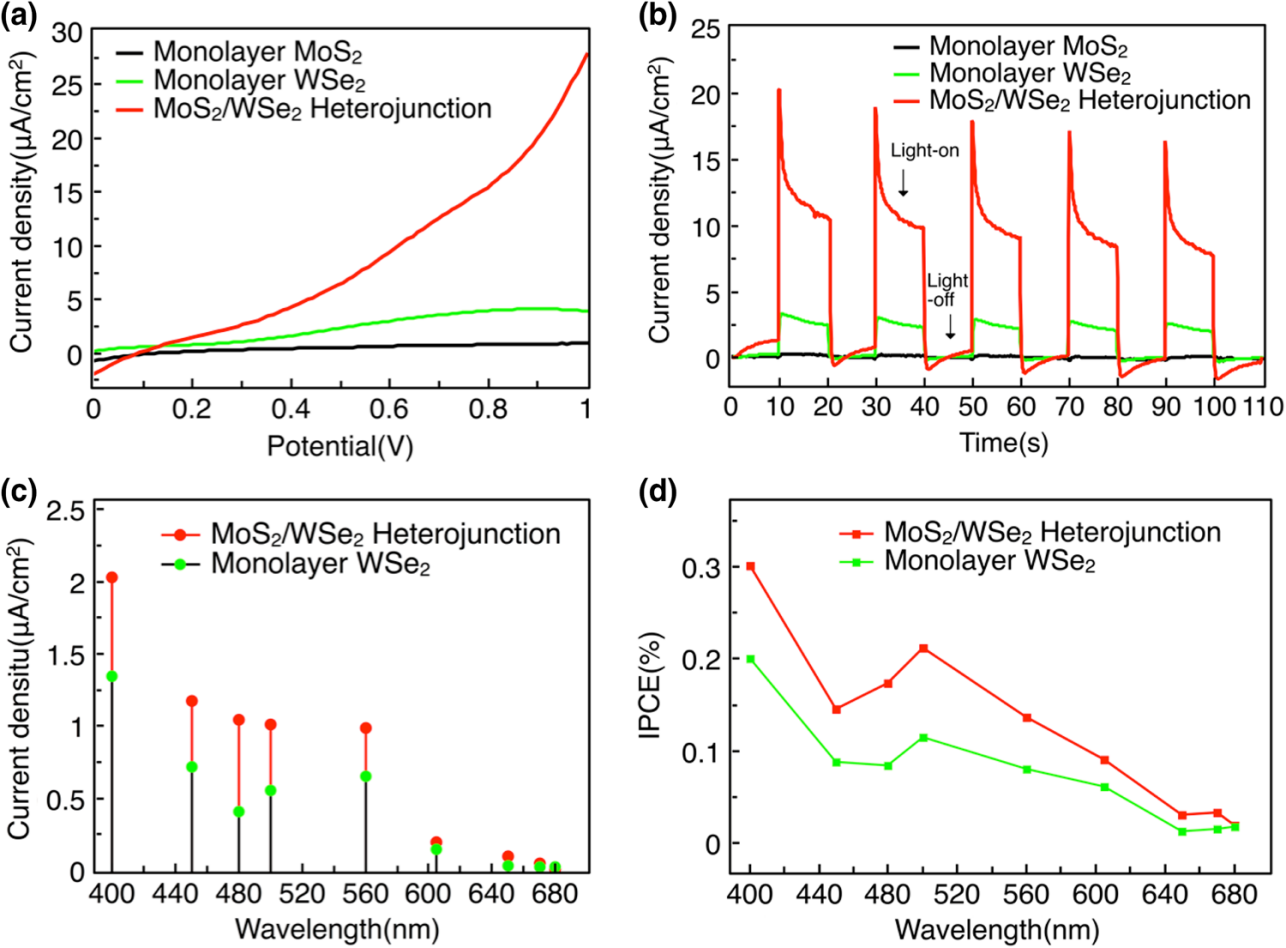
**a** PEC current density versus voltage (*J*–*V*) curves of MoS_2_/WSe_2_ heterojunction (red), monolayer WSe_2_ (green), and monolayer MoS_2_ (black) under the same white light illumination conditions. **b** Current response curves for the three samples under 1 V external bias with illumination an interval of 10 s. **c** PEC currents of monolayer MoS_2_/WSe_2_ heterojunction (red dots) and WSe_2_ (green dots) at different wavelengths. **d** IPCE of MoS_2_/WSe_2_ heterojunction (red dots line) and WSe_2_ (green dots line). (Color figure online)

The PEC current of single-layer WSe_2_, MoS_2_ and the heterojunction immersed in 0.5 mol L^−1^ Na_2_SO_4_ solution was measured under illumination with white light in order to investigate their PEC performance. The PEC current–voltage (*J*–*V*) curves of the above three materials under illumination are shown in Fig. 3a. MoS_2_ presented nearly no current response (Fig. 3a, black curve) owing to its intrinsic *n*-type characteristic. The Fermi level of the *n*-type semiconductor is higher than the electrochemical potential; hence, electrons will transfer from the semiconductor to the solution until the equilibration is achieved. After that, the bands of *n*-type semiconductor bend upward, which forms a barrier on the solid–liquid interface and blocks the transport of electrons [5]. This barrier layer hinders its catalytic activity as a PEC cathode. WSe_2_ presented a better PEC current response to the white light. A current density of 5 A cm^−2^ was recorded when the potential was swept to 1 V (Fig. 3a, green curve). As a *p*-type semiconductor, WSe_2_ [30, 38] is suitable for use as a PEC cathode. Due to its lower Fermi level, the bands of the *p*-type semiconductor bend downward when it is contacted with electrolyte solution. Without the barrier at the solid–liquid interface, the photo-generated electrons in WSe_2_ are easier to be driven toward the interface and move into the solution [5]. This bending facilitates the photo-generated electrons to reduce H^+^ and to transport more efficiently. The PEC current of the WSe_2_/MoS_2_ heterojunction (Fig. 3a, red curve) showed a much larger increase than the two aforementioned samples. A PEC current density of up to 28 μA cm^−2^ at 1 V bias was observed with the heterojunction, which is 5.6 times as large as that of WSe_2_. This provides initial evidence of the positive effect of the heterojunction on the PEC reaction.

The PEC current response curve was constructed for comparison with the visible-light response of the three aforementioned samples (Fig. 3b). The applied voltage was 1 V. All three samples exhibited a fast-optical response when the light was switched between the on and off states. In the light-on state, the decay of the PEC current is caused by the recombination of photo-generated electrons and holes. When illumination was interrupted, the photo-generated electrons at the surface suddenly vanished, and a gradually decline of the current was observed. Under the same illumination and bias conditions, the PEC current density of the MoS_2_/WSe_2_ heterojunction was three times as large as that of WSe_2_. This result is consistent with a former report that the heterojunction performed better than the single-material congeners in the PEC reaction [36, 39, 40].

### IPCE of Different Nanosheets

The incident photon-to-current conversion efficiency (IPCE) was measured and calculated for quantitative comparison of the light harvesting efficiency of the WSe_2_ and MoS_2_/WSe_2_ heterojunctions as a function of the wavelength. The IPCE (*η*) is defined as the ratio of the incident monochromatic photons converted to collected electrons and can be calculated by using Eq. 1

1 $$\eta = \frac{{_{{\mathop I\nolimits_{\text{ph}} }} }}{P} \times \frac{1240}{\lambda } \times 100\%$$

here *I*_ph_ (A) is the photocurrent, *P* (W) is the incident light power, and *λ* (nm) is the wavelength of light. The IPCE varied with different incident wavelength (*λ*). As shown in Fig. 3c, the PEC current density of the heterojunction and WSe_2_ was measured at wavelengths from 400 to 680 nm wavelength at bias of 1 V. The achieved PEC current density was smaller under monochromatic light than that obtained under white light given that the intensity of the monochromatic light decreased when filtered from the white light.

It was found that the heterojunction had a higher (Fig. 3c, red dots) current density in comparison with WSe_2_ (Fig. 3c, green dots) at all wavelengths. For example, the current density of the heterojunction at 480–500 nm was nearly twice as large as that of WSe_2_. The corresponding IPCEs of the heterojunction (red dot line) and WSe_2_ (green dot line) were calculated, as shown in Fig. 3d. Compared to the WSe_2_ counterpart, the MoS_2_/WSe_2_ heterojunction showed an obvious enhancement of the IPCE in the range of 400–680 nm. At 400 nm, the IPCE of the heterojunction exhibited a maximum value of 0.3%, which is 50% higher than that of WSe_2_. This is attributed to the highest absorption peak of WSe_2_ around 420 nm [29, 41], and the absorption rate in 400 nm is close to that in 420 nm. In addition, the WSe_2_/MoS_2_ heterojunction helps to increase the electron–hole separation efficiency. Therefore, the IPCE was largely improved with the heterojunction. The IPCE of the heterojunction was around 0.1–0.3% at 450–605 nm and decreased with a red-shift of the wavelength due to the reduced absorption of light at higher wavelength.

**Fig. 4:**
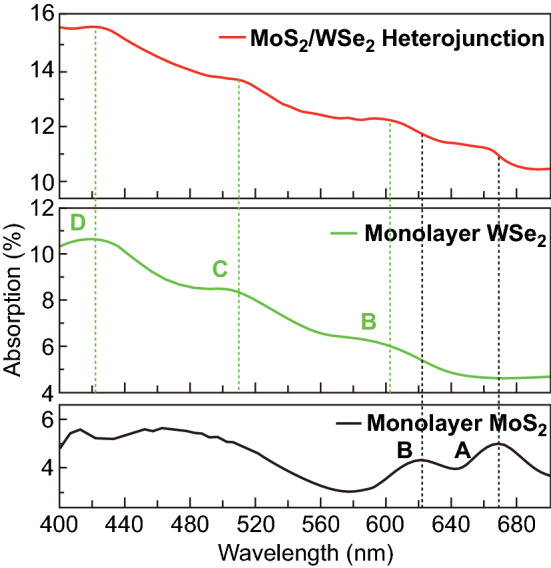
Absorption spectra of MoS_2_/WSe_2_ heterojunction (red curve), monolayer WSe_2_ (green curve) and monolayer MoS_2_ (black curve). (Color figure online)

The light absorption spectra of monolayer MoS_2_, WSe_2_ and MoS_2_/WSe_2_ heterojunction are shown in Fig. 4, which is consistent with former reports [29, 41–43]. The absorption spectrum of monolayer WSe_2_ (Fig. 4, green curve) possesses 3 peaks at ~ 420, 500, and 600 nm (labeled as D, C, B peak, respectively) in 400–700 nm. The light absorptivity is between 4.5 and 11% and decreases with wavelength. There are two absorption peaks in the absorption spectrum of monolayer MoS_2_ (Fig. 4, black curve) at ~ 620 and 670 nm (labeled as B and A peak, respectively). The light absorptivity is between 3.5 and 6%. The absorption spectrum of heterojunction (Fig. 4, red curve) is the sum of absorption spectrum of WSe_2_ and MoS_2_. The overall absorptivity is 1.5 times that of monolayer WSe_2_. Because the heterojunction consists of WSe_2_ and MoS_2_, it exhibits the absorption characteristics of monolayer WSe_2_ and MoS_2_. The peak surrounding at 500 nm in both IPCE of WSe_2_ and heterojunction is mainly due to the C absorption peak of monolayer WSe_2_, where more light was utilized. It is remarkable that the IPCE analysis of the heterojunction showed a small peak at 670 nm, compared to that of WSe_2_. This peak is attributed to the absorption of MoS_2_ at ~ 670 nm [42, 43]. Thus, the MoS_2_/WSe_2_ heterojunction was able to harvest more photons under 670 nm irradiation than WSe_2_. Meanwhile, the optical absorption of heterojunction can be improved by some means. For example, using nano-metal stripe to introduce surface plasmon resonances [44] or inserting a certain thickness of transparent electrode between heterojunction and back electrode to construct resonance back reflection [45] can further enhance light absorption.

Although monolayer WSe_2_ has three absorption peaks at 400–700 nm and its absorption gradually decreases from 400 to 700 nm, the peak at 600 nm is relatively weak. MoS_2_ has an enhanced absorption at ~ 670 nm. Therefore, the IPCE of the heterojunction was the highest at 400 nm and under excitation at the wavelengths of the two other peaks at 500 and 670 nm in the wavelength range of 400–680 nm. Overall, the improvement in the IPCE was mainly attributed to: (1) the *p*–*n* junction formed between MoS_2_ and WSe_2_, which increases the separation of the electron–hole pair; (2) broadening of the range of the solar spectrum absorbed by the stack of MoS_2_ and WSe_2_, leading to better utilization of the solar spectrum.

**Fig. 5:**
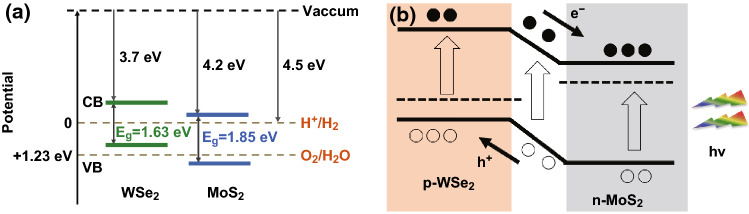
**a** Energy band position of monolayer MoS_2_ and WSe_2_ compared with redox potentials for water splitting. **b** MoS_2_/WSe_2_ heterojunction formed a type II band alignment. When illuminated, photo-generated electrons are transferred from valence band to conduction band and the built-in electric field helps to separate excitons

The energy band position of monolayer MoS_2_ and WSe_2_ compared with redox potentials of water splitting has shown in Fig. 5a. The relative band position between monolayer MoS_2_ and WSe_2_ is decided by their electron affinity. The electron affinity of WSe_2_ and MoS_2_ is *χ*_W_ ~ 3.7 eV and *χ*_M_ ~ 4.2 eV [46–48], respectively. For the heterojunction, the developed band alignment and built-in potential between *p*-WSe_2_ and *n*-MoS_2_ help to facilitate separation of the photo-generated exciton, as shown in Fig. 5b. Because MoS_2_ and WSe_2_ form a type II band alignment, the conduction band minimum (CBM) and valence band minimum (VBM) of WSe_2_ are higher than those of MoS_2_ [35, 36]. Upon irradiation, photons are absorbed and excitons are generated in single-layer WSe_2_ and MoS_2_. The photo-generated free electrons in the CBM of WSe_2_ can be transferred to the CBM of MoS_2_ owing to the large band offset between WSe_2_ and MoS_2_ [35, 36, 42]. Due to the built-in field existing across the stacking facet of WSe_2_ and MoS_2_, the photo-induced excitons then relax at the MoS_2_/WSe_2_ interface, driving more efficient separation [49]. The electrons then drift to the CBM of MoS_2_ while the holes drift to the VBM of WSe_2_. Hence, electrons are collected in the conduction band and transported to the solid–liquid interface. At the interface, the reduction reaction occurs at the surface of MoS_2_, in which H^+^ obtains electron to generate H_2_ (2H^+ ^+ 2e^− ^→ H_2_). It is noted that due to the atomic thickness of the ultrathin heterojunction, its built-in field would penetrate to the solid–liquid interface, thus having a positive potential to overcome the barrier on the interface and accelerate the redox reaction. In addition, the ultrathin heterojunction shortens the path for electron transport. The electrons could reach the interface immediately after separation. Therefore, compared with the single-layer material, the heterojunction leads to better utilization of light and furnishes more electrons for the reaction. As a result, a much higher PEC current and a greater IPCE were achieved with the MoS_2_/WSe_2_ heterojunction in comparison with WSe_2_ and MoS_2_.

## Conclusion

Monolayer *p*-type WSe_2_ and monolayer *n*-type MoS_2_ were stacked layer-by-layer to form a heterojunction by using a dry-transfer method. A WSe_2_/MoS_2_ heterojunction was fabricated to act as a miniaturized PEC cathode on the micrometer scale. In situ measurement was adopted to investigate the intrinsic PEC characteristics for comparison with the single-material cathode. The PEC current of the heterojunction was 5.6 times than that of the monolayer WSe_2_ under an external bias of 1 V under illumination with white light. The bilayer heterojunction also exhibited a 50% enhancement in the IPCE relative to the monolayer WSe_2_ within the visible light range of 400–680 nm. Derived from the type II band alignment formed between MoS_2_, WSe_2_ and the ultrathin thickness of the heterojunction, the heterojunction broadened the light harvesting range, improved the photo-induced exciton separation, and accelerated the carrier transport. The unique structure and superior PEC characteristics of the MoS_2_/WSe_2_ heterojunction suggest that it holds great promise as a photocathode for the HER with potential for efficient solar energy conversion applications.
